# Hydrogel Functionalized Polyester Fabrics by UV-Induced Photopolymerization

**DOI:** 10.3390/polym11081329

**Published:** 2019-08-10

**Authors:** Emanuela Lorusso, Wael Ali, Marcus Hildebrandt, Thomas Mayer-Gall, Jochen S. Gutmann

**Affiliations:** 1 Deutsches Textilforschungszentrum Nord-West ÖP GmbH, Adlerstr. 1, 47798 Krefeld, Germany; 2 Department of Physical Chemistry and Center of Nanointegration (CENIDE), University of Duisburg-Essen, Universitätsstr. 2, 45141 Essen, Germany; 3 Deutsches Textilforschungszentrum Nord-West gGmbH, Adlerstr. 1, 47798 Krefeld, Germany

**Keywords:** hydrogels, UV-photopolymerization, functional surfaces, PET fabrics

## Abstract

We address a strategy to graft hydrogels onto polyethylene terephthalate (PET) fabrics using different acrylate-based monomers. The hydrogel-modified fabrics were prepared by a two-step modification. To this end, double functional groups were firstly introduced onto the PET surface via an aminolysis reaction involving allylamine. The final grafted polymer networks were then obtained after UV-induced radical photopolymerization by varying acrylate monomer types in the presence of a cross-linker. After characterization, the resulting hydrogels showed different morphologies and abrasion resistance performances depending on their chemical nature. UV-photopolymerization is a fast and low-cost method to achieve technical fabrics with specific desired properties.

## 1. Introduction

**Scheme 1:**
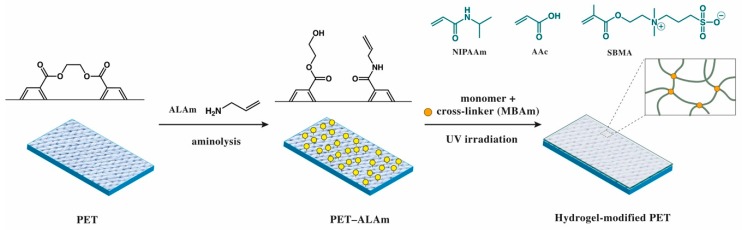
A synthesis route of the immobilization of hydrogels on polyethylene terephthalate (PET) fabrics by UV irradiation.

Surface modification of textile-based materials has attracted much attention in recent years due to its applicability in many interesting fields, including filtration, biomedical textiles, smart textiles, flame retardant fabrics, and textile catalysts [1,2,3,4,5,6]. In this context, polyethylene terephthalate (PET), also known as polyester, is the most commonly used fiber. The advantages of using PET as a carrier encompass the cost-effectiveness, which is a key feature for large-scale production, and its physical and chemical properties such as flexibility, stability, softness, lightness, elasticity, anti-wrinkling, etc. [6,7,8]. Due to its hydrophobic nature and the absence of functional groups, any permanent functionalization of PET implies a covalent linkage with the surface. Strategies to functionalize polyester fibers involved its pre-functionalization by means of adhesive polyelectrolytes rich in functional groups such as polyvinylamine (PVAm). Among other applications, these coatings have proven to be successful for metal removal from contaminated groundwater, presented antibacterial properties, and might act as a scaffold for a subsequent surface modification [9,10,11]. On the other hand, depending on the processes involved in the post-modification, obtaining a durable and homogeneous coating is still a challenging task since the nature and conformation of the polyelectrolytes very often influence the anchoring of some polymers. Aminolysis reactions using several amines are involved in the recycling of polyester fibers. During the process, the integrity of the fiber is compromised, yet, if conducted in well controlled conditions, this process offers the possibility of anchoring functional groups on the PET surface without drastically impacting the mechanical properties of the textile [10,12,13,14,15,16,17]. Scheme 1 shows the steps involved in the aminolysis reaction and the synthesis route. PET polymer chains are broken by reacting with an amine. This allows the amine to be covalently inserted in the polymer structure, generating functional groups and acting as a scaffold. The process allows for the choice of a reactant that best suits the subsequent surface modification process among a broad number of amines. Here we present a fast and easy method to covalently link hydrogels on PET fabrics using aminolysis of the PET polymer chains, followed by UV-induced radical photopolymerization of acrylate monomers. Hydrogels are cross-linked, three dimensional, hydrophilic polymer networks that swell when brought into contact with water but do not dissolve. Depending on the chemical nature of the polymers and the synthetic routes, hydrogels are able to respond to external stimuli that induce changes in their conformation and function [18]. In particular, poly *N*-isopropylacrylamide (PNIPAAm) is a well-known thermo-responsive polymer that exhibits a lower critical solution temperature (LCST) at around 32 °C in aqueous solution. Hence, PNIPAAm hydrogels induce a reversible change in volume resulting in switchable surface properties. Very few results have been reported on the grafting of smart polymeric hydrogels on textiles. Chen et al. have grafted PNIPAAm hydrogel onto polypropylene nonwoven fabrics and PET film using argon plasma pretreatment followed by photo induced surface graft polymerization [19]. Recently, PNIPAAm hydrogel-modified PET textiles have also been prepared via photo-grafting PNIPAAm onto PET surfaces that have been functionalized through carboxylation and hydrolysis process. Such smart and highly hydrated surfaces were found to be very effective in many application fields, including antifouling systems and oil/water separation strategies [9,20,21,22,23]. For these reasons, extending these approaches onto PET fabrics is desirable in order to achieve textiles-based stimuli responsiveness or smart fabrics for medical applications, comfort improvement in clothing, and switchable membranes.

## 2. Materials and Methods

### 2.1. Materials

All experiments were carried out using polyester fabrics (wfk-Testgewebe GmbH, Brüggen-Bracht, Germany) with a surface weight of 170 g/m^2^ and yarn count of 295/295 dtex. The PET fabrics were extracted with ethanol-water (1:1) and then with petroleum ether to remove all possible contaminations. Allyl amine (ALAm, 98%), *N*-Isopropylacrylamide (NIPAAm, 99%), acrylic acid (AAc, 99%), *N*,*N*′-methylenebisacrylamide (MBAm, 99%), and [2-(Methacryloyloxy)ethyl]dimethyl-(3-sulfopropyl)ammonium hydroxide (SBMA, 95%) were obtained from Merck (USA, Sigma-Aldrich formerly). The inhibitor hydroquinone monomethyl ether (MEHQ) and possible oligomers content were removed from the monomers solutions before the polymerization, using an inhibitor remover purchased from Merck (USA). Ethanol (99% denatured with 1% methyl ethyl ketone (MEK) Technical grade) was purchased from AppliChem GmbH (Darmstadt, Germany). Water was deionized using a Milli-Q system (Millipore, Burlington, MA, USA).

### 2.2. Surface Modification of PET Fabrics with Hydrogels

In order to modify PET fabrics with hydrogels, the original fabrics were cut into circular samples with a diameter of 4 cm. Double functional groups were firstly introduced onto the PET surface via an aminolysis reaction involving ALAm. The modification occurred in a flask filled with a 15% *v/v* ALAm in ethanol for 2 h. The samples were then extensively rinsed with water and left to dry. After the pre-modification, UV-induced radical photopolymerization was used to grow the hydrogel network on PET fabrics. 10% *w/v* aqueous solutions of SBMA, AAc, and NIPAAm were prepared adding MBAm (5% wt. in respect to the monomer concentration) as a crosslinker (Scheme 1).

The pre-treated PET fabrics were immersed in the monomer solution and left to equilibrate for several hours. The samples were removed from the solutions, placed on a paper wipe to remove excess water, and put in a well-plate. The well-plate was then positioned inside an in-house built chamber. This consisted of a metal box with a quartz window facing the incoming radiation. The chamber was equipped with a gas inlet and a purge connector in order to evacuate it properly with nitrogen gas. The chamber was inserted in a cabin under the UV lamp (UVA cube 2000, Dr. Hoenle AG, Germany) within a distance of 10 cm from the samples with power of irradiance equal to 160 mW/cm^2^. The samples were irradiated for 5 min according to a pre-test carried out for bulk hydrogels. After irradiation, the samples were washed with water to remove unbound moieties and left to dry.

### 2.3. Characterization

Fourier-transform infrared spectra (FTIR) were acquired in attenuated total reflectance (ATR) mode using IR Prestige (Shimadzu, Kyoto, Japan). A golden gate, diamond crystal (Specac, Orpington, UK), with a resolution of 4 cm^−1^ was used for the attenuated total reflection. The morphology of the hydrogel-modified PET fabrics was observed using scanning electron microscopy (SEM, S-3400 N II, Hitachi High Technologies Europe GmbH, Tokyo, Japan). The samples were immersed in deionized water at room temperature for 1 h to reach the swelling equilibrium and then were frozen in liquid nitrogen. The frozen PET fabrics were freeze-dried at −50 °C for 24 h using a freeze dryer system (Christ Alpha 1-4, Osterode, NI, Germany). Before the measurement, the surfaces of the modified PET fabrics were sputtered with gold in a vacuum for 4 min using a sputter coater (Quorum Emitech K500X, Ashford, Kent, UK). The surface topography and phase of the modified fabrics were also characterized using scanning probe microscopy (SPM). The measurements were conducted with Agilent Technologies 5500 beam deflection SPM using nanosensor tapping-mode cantilevers with silicon tips (AC frequency 146−236 kHz, length 225 ± 10 μm, width 38 ± 7.5 μm, thickness 7 ± 1 μm, force constant 21−98 N/m, tip height 10–15 μm). The resolution of all images was set at 1024 data points with a scanning speed of 0.5 lines per second. All captured images (5 µm × 5 µm) were processed by Gwyddion software, and the roughness profiles *R*q (root mean square (RMS)) of the whole image at maximum resolution were obtained via row/column statistical analysis.

Abrasion tests were performed using a Martindale apparatus with a pressure of 12 kPa. The diameter of the sample was 4 cm with an abrasive exposed diameter of 3 cm. The surface properties of the PET fabrics were evaluated by measuring the time required for deionized water droplets (20 µL, methylene blue staining) to penetrate into the fabrics (26 mm diameter) (wettability test). The static water contact angle was also measured at room temperature by employing the sessile drops method.

## 3. Results and Discussion

The monomers were chosen to explore different types of temperature-responsive solution behaviors. This may lead to the synthesis of coating exhibiting interesting morphologies.

**Figure 1:**
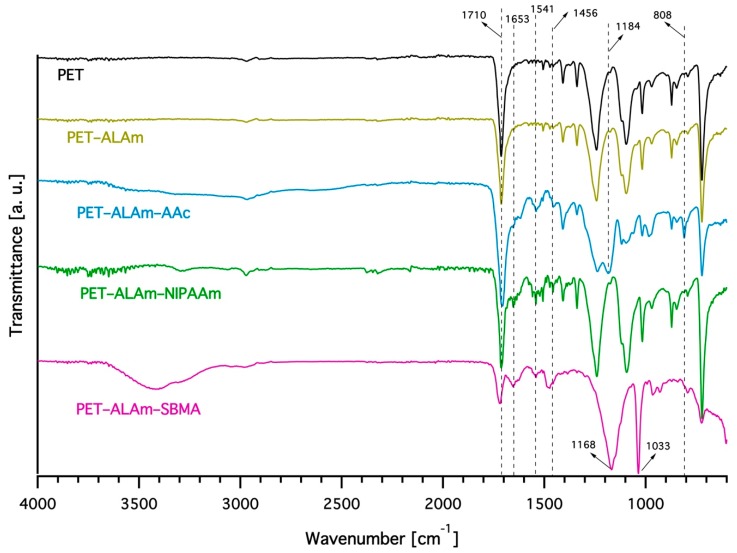
Attenuated total reflectance-fourier-transform infrared (ATR-FTIR) spectra of pristine PET, PET modified with allylamine (PET-ALAm), and hydrogel modified PET fabrics.

Qualitative characterization was conducted to assess the efficiency of the functionalization. Figure 1 reports the ATR-FTIR spectra of the hydrogel modified polyester fabrics. There is no appreciable difference between PET and PET-ALAm, since the signals of residual double bonds or amines on the surfaces were masked by the bulk polymer signals of PET. After subsequent modification, the hydrogels consisted in a thick and uniform layer on the fabric, therefore the signals related to the bulk PET were attenuated by the new coating. All spectra present a signal at 1710 cm^−1^ attributable to the C=O stretching of the carbonyl group. For PET-ALAm-AAc, a band in between 3000–3500 cm^−1^ reveals an O-H stretching belonging to PAAc hydrogel, and the band at 1184 cm^−1^ is due to the –(C–O)H stretching mode of PAAc. The band at 808 cm^−1^ belongs to the CH_2_ rocking mode of PAAc. PET-ALAm-NIPAAm exhibits two absorbance bands at 1653 cm^−1^ and 1541 cm^−1^, attributed to the stretching of secondary amide in PNIPAAm. The band in the range of 3200–3400 cm^−1^ belongs to the N–H stretching, while the small band at 1456 cm^−1^ is due to the C–N stretching band. The band at 3417 cm^−1^ in PET-ALAm-SBMA corresponds to the O–H stretching band of water and resulted from the interaction of moisture present in the air with the charged sites of SBMA in the hydrogel matrix. The bands at 1033 cm^−1^ and 1168 cm^−1^ are assigned to the sulfonyl group (–SO_3_).

**Figure 2:**
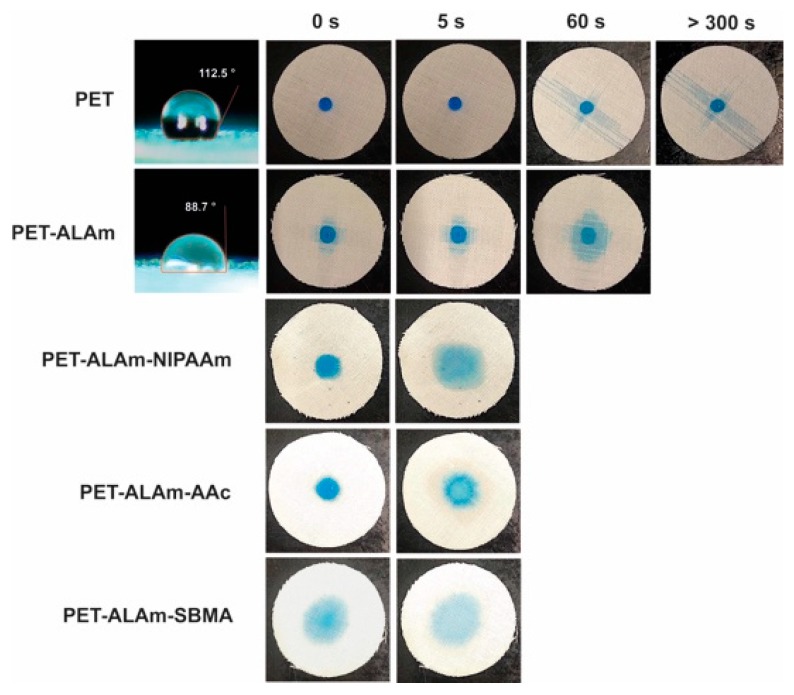
Photographs show wettability measurements on the pristine and modified PET fabrics using both contact angle measurements (sessile drop) and drop penetration tests.

The hydrophilic/hydrophobic properties of the pristine PET and PET-ALAm were investigated by sessile drop technique. Figure 2 shows that PET fabrics undergo a reduction of the contact angle from ~113° to 89° after the aminolysis process, indicating the successful introduction of the double bonds onto the surface. A decrease of the hydrophobicity of the PET fabrics can be attributed to the presence of OH groups after the aminolysis process, as shown in Scheme 1. Reductions of the droplet penetration time into the fabrics indicates an increase in hydrophilicity of the surface. It can be observed that pristine PET shows a capillary effect, while water droplets circularly penetrate into the PET-ALAm fabrics, which may indicate a homogeneous pre-modification of the PET with ALAm.

In the case of hydrogel modified PET fabrics, water droplets penetrate the fabrics in less than 5 s. In particular, the surface of PET-ALAm-SBMA samples is the most hydrophilic, with a penetration time of less than 1 s.

**Figure 3:**
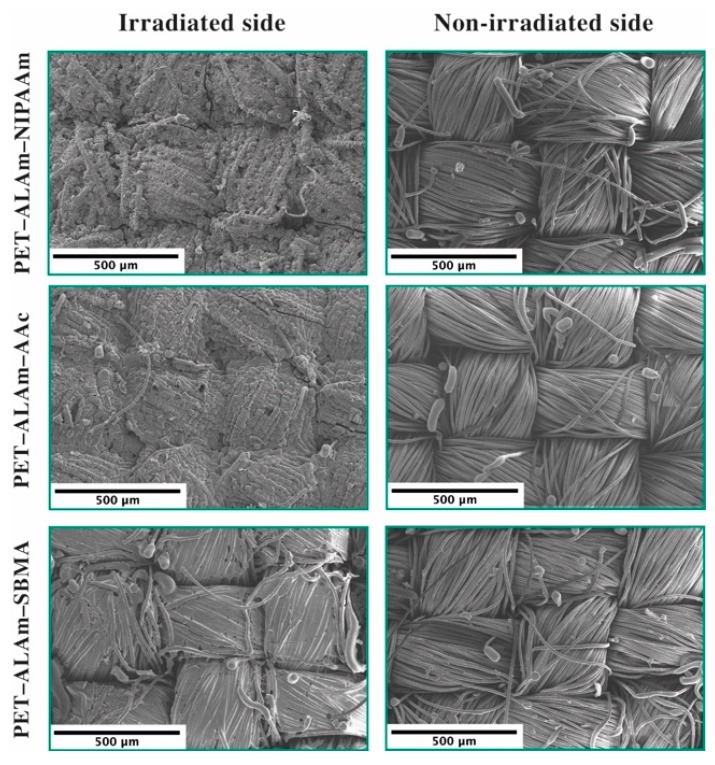
SEM morphologies of irradiated side and non-irradiated side of hydrogel modified PET fabrics.

The advantage of hydrogel synthesis using UV-irradiation lies in the reversible features imparted to the PET fabric. SEM images of hydrogel modified PET after a freeze-drying cycle are reported in Figure 3. The set shows two images belonging to different sides of the same sample. After immersion in the hydrogel solutions, the samples were impregnated by capillarity. However, if an area was not exposed to the UV light, the modification did not occur. Therefore, on the irradiated side, the samples showed a successful modification of the PET fabric, while modification did not occur on the non-irradiated side. This would offer the possibility to modify both sides of the textile with gels exhibiting opposite properties, e.g., oleophobic/hydrophobic, or gels that are sensitive to different stimuli.

**Figure 4:**
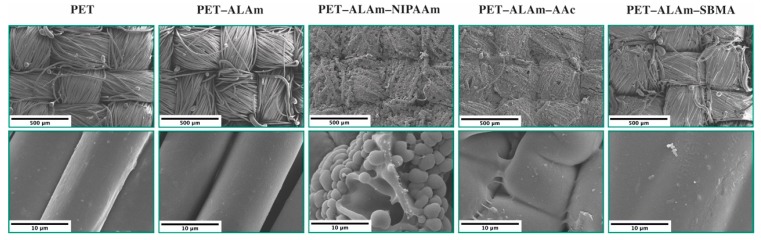
SEM morphologies of pristine PET, PET modified with allylamine (PET-ALAm), and hydrogel modified PET fabrics.

Figure 4 shows the magnified SEM images of the hydrogel modified fabrics. Freeze drying cycles of the swollen hydrogel were crucial in order to capture the original conformation of the hydrogels after swelling. The pre-treatment with allylamine did not alter the appearance of the PET fibers. Instead, after functionalization, the resulting surfaces exhibited different morphologies. PET-ALAm-NIPAAm hydrogel formed a granular structure whereas PET-ALAm-SBMA appeared as a homogeneously distributed coating. This is due to the characteristic behavior of the monomer solutions at different temperatures. As PNIPAAm exhibits a LCST at around 32 °C, the forming hydrogel undergoes a phase transition to a shrunken dehydrated state under irradiation when the temperature is higher than the LCST of PNIPAAm [24,25,26]. As a consequence, crosslinked PNIPAAm generates agglomerates of microgel particles instead of hydrogel networks. In contrast, PSBMA is an upper critical solution temperature (UCST) type responsive polymer. Therefore, when SBMA hydrogel is formed, high temperatures promote a swollen hydrated state of the hydrogel [22,27,28].

Additionally, acrylic acid solutions do not exhibit any critical solution temperature in the operating working range. The hydrogel layer on PET-AlAm-AAc appeared in a swollen state, showing a compact aggregate of polymer chains.

**Figure 5:**
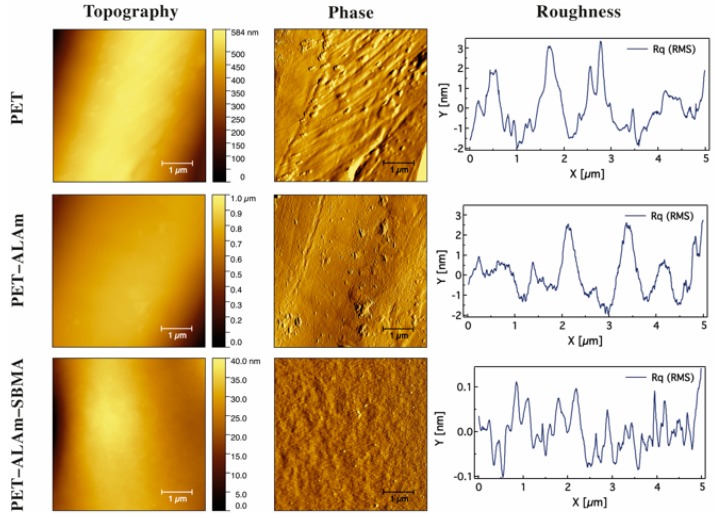
Scanning probe microscopy (SPM) images representing topography and phase of hydrogel modified PET fabrics.

Figure 5 reports the SPM images of the hydrogel grafted on PET. Pristine PET, Allylamine grafted PET, and PET modified with the SBMA hydrogel are shown, respectively. The images do not show any change in roughness after the aminolysis step in agreement with the observations of SEM. In contrast, after modification with SBMA hydrogel, the RMS profile presents a lower surface roughness in comparison with that of PET-ALAm or pristine PET. It should be noted that the SPM images were recorded without the freeze-drying process.

**Figure 6:**
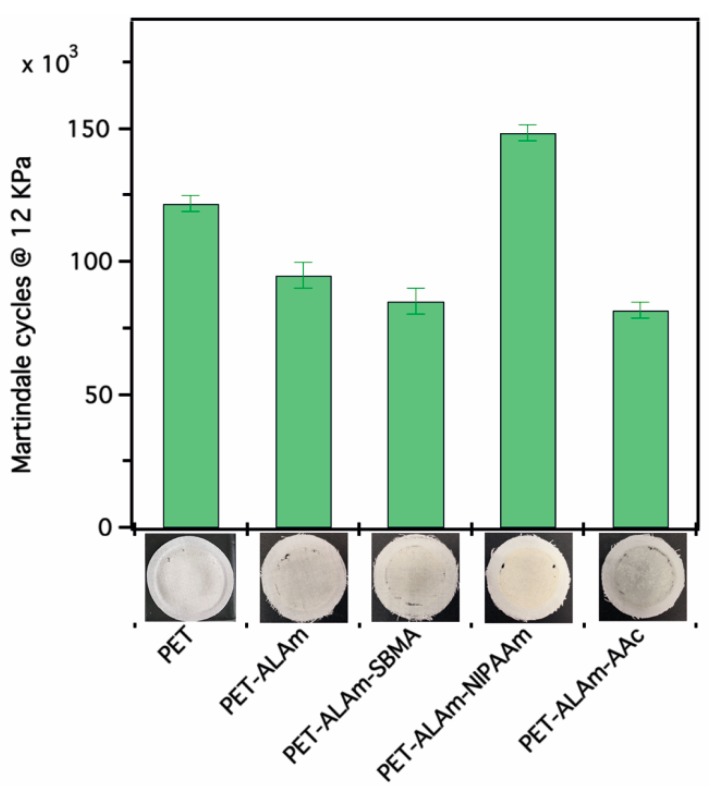
Martindale cycles of PET and hydrogel coated PET samples.

We investigated the effect of abrasion on the functionalized PET samples. The abrasion resistance tests were performed using Martindale cycles. Results are reported in Figure 6. After each functionalization step, the fibers lost their integrity due to the covalent addition on the amine during the aminolysis reaction. This led to a reduction of the abrasion resistance, which is accentuated by the exposure to UV radiations in the polymerization step. This reduction is evident in PET-ALAm, PET-ALAm-SBMA, and PET-ALAm-AAC. On the other hand, PET-ALAm-NIPAAm showed improved abrasion resistance performances. This was a consequence of the microgel particle formation after UV polymerization that seemed to reduce friction between the sample itself and the standard abrasive surface employed in the measurement system, acting as micro-cushions [29]. Further studies will involve tensile strength tests in order to investigate how aminolysis affects the mechanical properties of the fibers.

## 4. Conclusions

In summary, we successfully grafted PNIPAAm, PSBMA and PAAc hydrogel networks onto PET fabrics by means of pre-functionalization with allylamine, followed by UV polymerization. Allylamine reacted with PET by aminolysis and served as a scaffold for the covalent bonding of the hydrogel networks. Coatings with different morphologies were achieved due to the different thermo-responsive behavior in solution of the selected monomers. PET-ALAm-SBMA and PET-ALAm-AAc showed comparatively low abrasion resistance. Although PET-ALAm-AAc presented agglomeration of polymer chains, the structure did not lead to any improvement of the abrasion performances. PET-ALAm-NIPAAm consisted of microgel particles grafted on PET fabrics as a consequence of NIPAAm solutions exhibiting LCST. The microgel particles act as a cushion, thus limiting friction and reducing abrasion. This simple and low-cost method is promising for the textile industry and might be applied using a wide range of polymers in order to achieve smart and technical fabrics with properties that meet any desire.
